# Reverse-Wire TEVAR for Subacute Type B Aortic Dissection with Severe True-Lumen Collapse: A Case Report

**DOI:** 10.3390/life15121879

**Published:** 2025-12-09

**Authors:** Andrada Bogdan, Mircea Robu, Elena Nechifor, Aida Badea, Maria Sabina Safta, Alexandru Zaman, Andrada Guță, Bogdan Gaşpar, Gabriel Gorecki, Horațiu Moldovan

**Affiliations:** 1 Faculty of Medicine, Carol Davila University of Medicine and Pharmacy, 050474 Bucharest, Romania; bogdan_andrada@ymail.com (A.B.); mariasabinasafta@gmail.com (M.S.S.); bogdan.gaspar@umfcd.ro (B.G.); h_moldovan@hotmail.com (H.M.); 2 Department of Cardiovascular Surgery, Clinical Emergency Hospital Bucharest, 014461 Bucharest, Romania; nechiforelena21@yahoo.com (E.N.); aidafirtade@gmail.com (A.B.); alexandrusebastianzaman@gmail.com (A.Z.); andrada.c.guta@gmail.com (A.G.); 3 Department of General Surgery, Clinical Emergency Hospital Bucharest, 014461 Bucharest, Romania; 4 Faculty of Medicine, Titu Maiorescu University, 040441 Bucharest, Romania; 5 Academy of Romanian Scientists, 54, Spl. Independenței, 050711 Bucharest, Romania

**Keywords:** type B aortic dissection, subacute TBAD, TEVAR, hybrid aortic repair, malperfusion syndrome

## Abstract

Type B aortic dissection (TBAD) requires management tailored to the disease phase and clinical presentation, with the subacute period representing a favorable window for endovascular intervention due to improved procedural safety and remodeling potential. We report the case of a 38-year-old male with hypertension, dyslipidemia, and bicuspid aortic valve disease who presented one month after symptom onset with persistent chest pain and progressive bilateral lower-limb numbness. Clinical examination suggested early spinal cord ischemia, while laboratory tests demonstrated acute hepatic and renal dysfunction. CT angiography revealed a subacute TBAD with a markedly expanded false lumen and near-complete compression of the true lumen, resulting in visceral, renal, and potential spinal malperfusion. Given the high-risk anatomy and evolving organ dysfunction, a staged hybrid strategy was undertaken. A left carotid–subclavian bypass was performed to secure proximal landing for endovascular repair, followed the next day by thoracic endovascular aortic repair (TEVAR) using two thoracic stent grafts. Postoperative recovery was favorable, with rapid resolution of neurological symptoms and normalization of hepatic and renal parameters, allowing discharge on postoperative day seven. This case underscores the importance of early recognition of malperfusion and timely hybrid intervention in subacute TBAD with severely compressed true lumen, demonstrating excellent early clinical outcomes.

## 1. Introduction

Type B aortic dissection (TBAD), accounting for 25–40% of all aortic dissections, is defined by involvement of the aorta distal to the left subclavian artery [1]. Its management is shaped by two essential factors: the temporal phase of the disease and the presence of complications. According to the International Registry of Aortic Dissection, TBAD progresses through hyperacute, acute, subacute, and chronic phases, each with distinct therapeutic implications due to the progressive stiffening of the dissection flap and declining remodeling potential [2,3,4].

While optimal medical therapy remains the cornerstone of treatment for uncomplicated TBAD, thoracic endovascular aortic repair (TEVAR) has emerged as the gold-standard therapy for complicated TBAD, demonstrating superior early outcomes compared with open surgical repair [5,6,7,8]. The timing of intervention is critical. Increasing evidence shows that the subacute phase (approximately 15–90 days after symptom onset) represents the optimal window for TEVAR, offering an advantageous balance between procedural safety and the potential for favorable aortic remodeling [3,9,10].

Patients who develop early indicators of adverse evolution—such as refractory hypertension, persistent or recurrent pain, early aortic expansion, dynamic or static malperfusion, or imaging signs of impending rupture or false-lumen pressurization—are particularly suitable candidates for early TEVAR, even as they transition into the subacute phase [2,4].

Thus, early identification of complications and timely endovascular intervention—preferably within the acute–subacute window—remain key determinants of prognosis in the management of complicated TBAD.

## 2. Case Report

We report the case of a 38-year-old male patient with a known history of hypertension, dyslipidemia, and a mildly ectatic ascending aorta associated with a bicuspid aortic valve and moderate aortic regurgitation. Approximately one month prior to presentation, the patient experienced severe anterior chest pain, which partially subsided but did not completely resolve despite self-administered analgesic medication. While still experiencing persistent thoracic discomfort, he subsequently developed bilateral lower-limb numbness, prompting his presentation to our emergency department.

Upon arrival in the emergency department, the clinical and neurological examination revealed a hemodynamically and respiratory stable patient. Blood pressure measurements showed an interarm differential, with 150/80 mmHg in the right arm and 125/50 mmHg in the left arm, while the heart rate was 110 bpm, regular. Peripheral oxygen saturation on room air was 99%. Palpation identified a slightly diminished left radial pulse compared with the right, while femoral pulses were present and symmetrical.

Although the lower limbs were well perfused, without clinical signs of peripheral ischemia, the patient reported progressive numbness and a sensation of heaviness in both legs, accompanied by intermittent balance and coordination difficulties. Neurological examination confirmed reduced fine touch discrimination and diminished position sense (proprioception) in the lower extremities, findings consistent with early signs of spinal cord ischemia.

At presentation, laboratory analysis confirmed multiorgan involvement consistent with systemic hypoperfusion. Renal function was severely impaired, with serum creatinine of 6.0 mg/dL, estimated glomerular filtration rate < 10 mL/min/1.73 m^2^, and markedly elevated blood urea of 145 mg/dL. Mild metabolic acidosis was present (pH 7.31, base deficit −6 mmol/L), accompanied by slightly increased lactate (2.8 mmol/L). Liver enzymology was suggestive of hepatocellular injury, with AST 86 U/L and ALT 92 U/L, approximately two times above the upper limit of normal. Coagulation parameters showed no evidence of consumptive coagulopathy (INR 1.13; platelets 236,000/μL). Inflammatory markers were moderately elevated (CRP 18 mg/L), most likely secondary to hypoperfusion rather than infection.

Transthoracic echocardiography performed at presentation demonstrated a left ventricle with a telediastolic diameter at the upper limit of normal, measuring 56 mm, with preserved global systolic function. The right ventricle measured 35 mm in the basal view, exhibiting both normal radial and longitudinal contractility. The ascending aorta measured 43 mm, while the aortic root at the level of the sinuses of Valsalva measured 40 mm. The aortic valve appeared bicuspid, associated with minimal aortic regurgitation. At the level of the aortic arch, in proximity to the brachiocephalic trunk, the aortic diameter was 41 mm. No additional valvular abnormalities were noted, and no pericardial effusion was identified.

**Figure 1:**
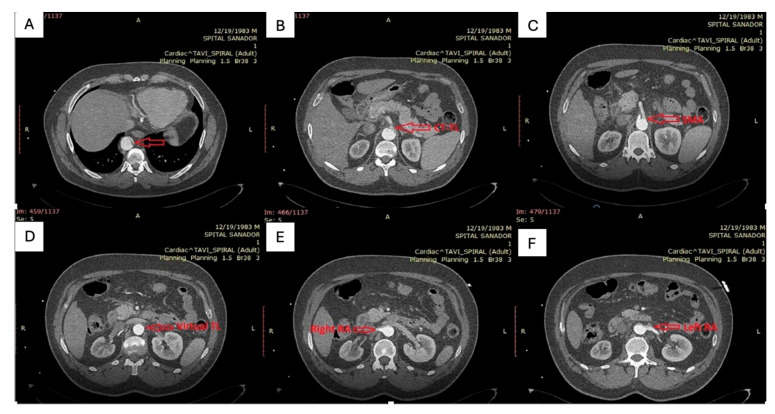
(**A**): Distal thoracic aorta with severely compressed true lumen; (**B**,**C**): Celiac trunk and superior mesenteric artery originating from the severely compressed true lumen; (**D**): severely compressed true lumen at the level of abdominal aorta; (**E**,**F**): Right renal artery perfused by the false lumen and left renal artery perfused by the true lumen.

Contrast-enhanced CT angiography (Figure 1) of the chest, abdomen, and pelvis revealed a type B aortic dissection originating immediately distal to the emergence of the left subclavian artery. Along the descending thoracic and abdominal aorta, the false lumen was markedly dilated, clearly dominating the aortic cross-section, while the true lumen appeared severely compressed and crescent-shaped, in some segments nearly effaced. At the thoracic level, the enlarged false lumen exerted visible compressive effect on multiple intercostal arteries, with potential implications for spinal cord perfusion.

At the abdominal level, the celiac trunk and the superior mesenteric artery arose from the extremely narrowed true lumen, which remained patent but critically reduced in caliber. The right renal artery was perfused from the false lumen, whereas the left renal artery originated from the true lumen, consistent with asymmetric renal perfusion. The dissection flap extended distally to involve both common iliac arteries, with persistence of the true–false lumen configuration into the iliac bifurcation.

**Figure 2:**
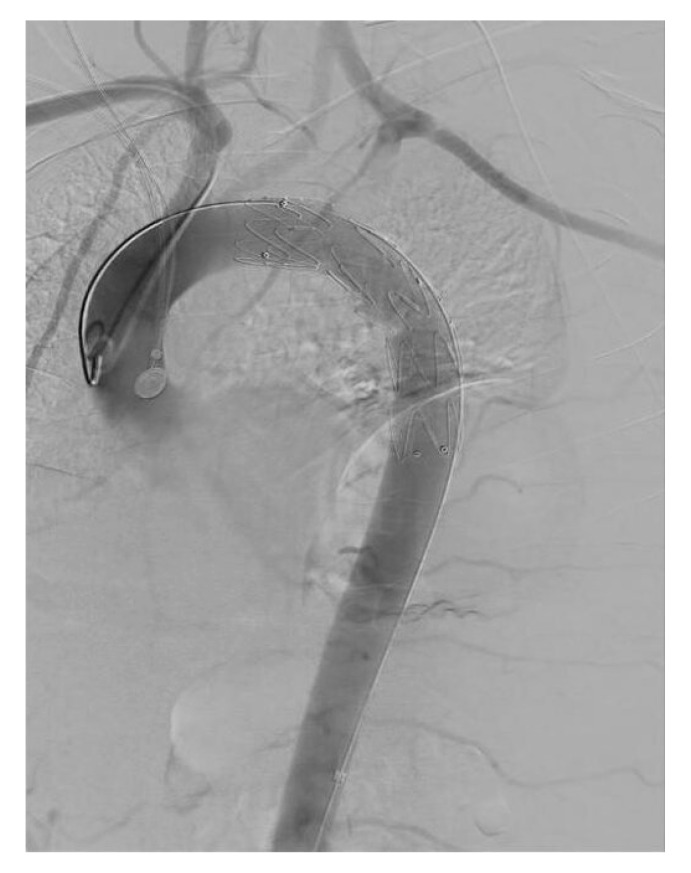
Angiographic representation of carotid-subclavian bypass.

**Figure 3:**
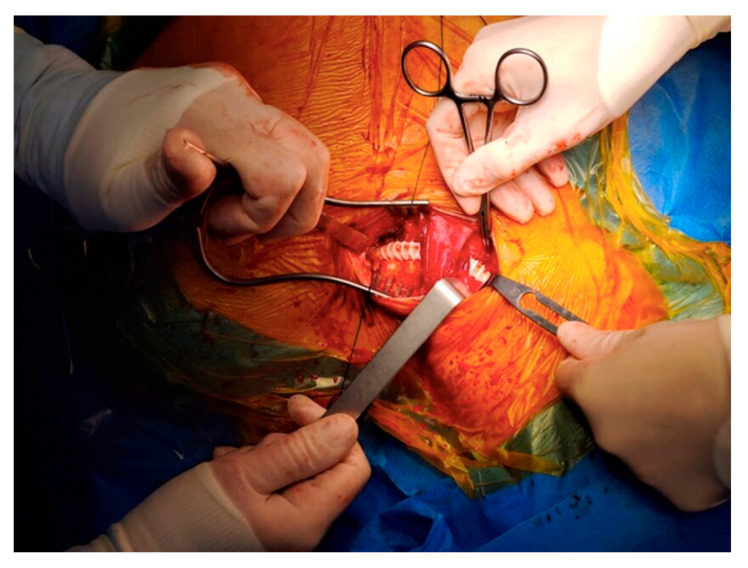
Intraoperative aspect of the carotid-subclavian bypass.

After reviewing the CT angiography findings, a two-stage hybrid approach was planned. The first stage consisted of a left carotid–subclavian bypass performed through a single supraclavicular incision, using an 8 mm reinforced vascular graft, followed by ligation of the left subclavian artery. The angiographic appearance is shown in Figure 2, and the intraoperative view in Figure 3. On the following day, the second stage was completed with thoracic endovascular aortic repair (TEVAR) using a Valiant Captivia thoracic endoprosthesis.

**Figure 4:**
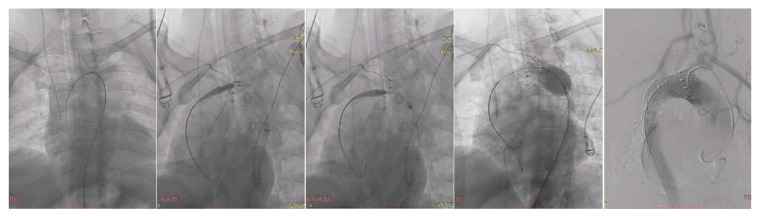
TEVAR steps illustration.

Under general anesthesia, vascular access was obtained via the right radial artery using a 5F sheath and via the right common femoral artery through a surgical cut-down with placement of a 7F sheath. The procedural steps are illustrated in Figure 4. A 5F pigtail catheter introduced through the right radial approach was advanced into the aortic arch to confirm accurate positioning within the true lumen using pressure waveform analysis. Through the right femoral access, a pigtail catheter was advanced into the ascending aorta, allowing visualization of the origins of the supra-aortic vessels by diagnostic aortography. A single-body wire technique was then performed, followed by wire reversal, with the C-arm positioned in a right posterior oblique projection to optimize delineation of the aortic arch anatomy.

Advancement of the endovascular system across the aortic arch was technically challenging due to its pronounced angulation.

The first endograft deployed was a Valiant Captivia thoracic stent graft (Medtronic Inc., Santa Rosa, CA, USA), positioned at the proximal descending thoracic aorta to secure sealing distal to the carotid–subclavian bypass. Its conformable proximal configuration allowed accurate apposition within the arch curvature and minimized the risk of bird-beak formation. Balloon molding of the proximal landing zone was subsequently performed using an E-Xpand balloon.

After achieving proximal stability, a second device—an E-vita Thoracic stent graft (Jotec GmbH/CryoLife Inc., Hechingen, Germany)—was advanced into the descending thoracic aorta and deployed across the segment of severe true-lumen compression. The stent system was selected for its higher radial force and scaffold stability, favoring true-lumen re-expansion in the context of false-lumen dominance. Adequate overlap between the prostheses was ensured, while preserving the origin of the celiac trunk. Completion angiography confirmed correct positioning of both stent grafts without evidence of endoleak. The origin of the celiac trunk was preserved, and at this anatomical level the true lumen reconstituted its flow, as intended. Completion angiography demonstrated correct positioning of both grafts without evidence of endoleak. All devices were then removed, and the femoral arteriotomy was surgically closed.

Device selection was driven by the specific anatomical and biomechanical characteristics of the aorta. For the proximal landing zone, we selected a Valiant Captivia thoracic endoprosthesis, as its conformable proximal configuration provides secure sealing in the arch curvature and minimizes the risk of bird-beak formation or apposition gaps, especially in the setting of a carotid–subclavian bypass. Conversely, for the distal thoracic segment, we opted for an E-VITA JOTEC stent graft, which exhibits higher radial force and greater scaffold stability along the compressed true lumen. These properties are advantageous in cases of near-complete lumen collapse, where the false lumen dominates the cross-sectional geometry and external compression may impede device expansion. The sequential use of these two devices therefore balanced arch conformability and distal structural support, facilitating uniform re-expansion of the true lumen without compromising visceral branch perfusion.

Following TEVAR, a rapid improvement of organ-specific markers was observed. Within the first 48 h, serum creatinine decreased from 6.0 mg/dL to 3.4 mg/dL, with a further decline to 1.8 mg/dL on day 5. Blood urea dropped from 145 mg/dL to 78 mg/dL, then to 47 mg/dL at discharge. Hepatic cytolysis regressed significantly: AST decreased to 39 U/L, ALT to 48 U/L by day 3, indicating resolution of ischemic hepatopathy. Lactate normalized (<2 mmol/L) and acid–base balance returned to physiological range.

**Figure 5:**
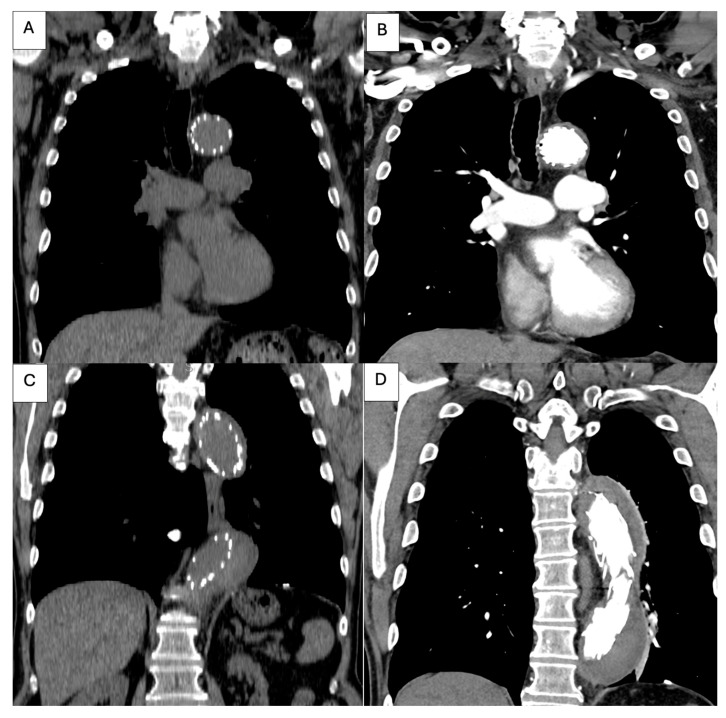
Post-procedural thoracic CT angiography. (**A**): Coronal reconstruction demonstrating the proximal landing zone of the thoracic endograft immediately distal to the left subclavian artery, with satisfactory device apposition to the aortic wall; (**B**): Coronal view showing the proximal segment of the stent graft within the descending thoracic aorta, with complete thrombosis of the false lumen and restored patency of the true lumen; (**C**,**D**): Coronal reconstructions depicting the endograft extending along the descending thoracic aorta, with complete false-lumen thrombosis and uniform expansion of the true lumen, confirming adequate endovascular sealing and favorable early remodeling.

**Figure 6:**
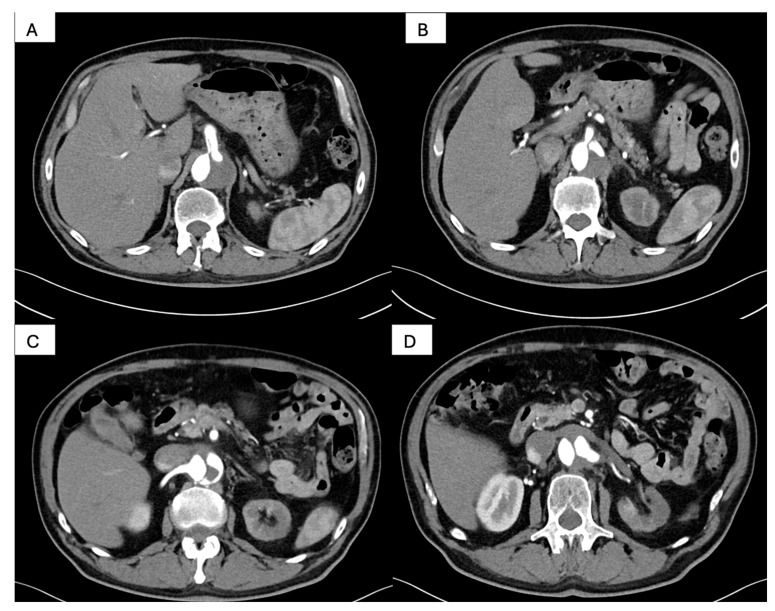
Postprocedural abdominal CT angiography. (**A**): Axial reconstruction demonstrating the celiac trunk perfused through the true lumen, with persistence of the dissection flap and retrograde opacification of the false lumen; (**B**): Axial view showing the superior mesenteric artery originating from the true lumen, with preserved antegrade visceral perfusion; (**C**): Axial reconstruction illustrating the right renal artery arising from the true lumen, with homogeneous contrast enhancement and satisfactory renal perfusion; (**D**): Axial view demonstrating the left renal artery filling retrogradely from the false lumen, which remains perfused via the celiac axis, confirming asymmetric renal arterial supply.

Postprocedural CT angiography (Figure 5 and Figure 6) demonstrated favorable remodeling of the thoracoabdominal aorta. At the thoracic level, the thoracic endografts exhibited complete sealing of the proximal entry tear with uniform re-expansion of the true lumen and full thrombosis of the false lumen, extending along the descending thoracic aorta. Distally, the celiac trunk was perfused through the true lumen, although the residual dissection flap maintained a retrograde opacification of the false lumen. The superior mesenteric artery originated from the true lumen with antegrade contrast enhancement, indicating preserved splanchnic perfusion. Renal perfusion was asymmetric: the right renal artery arose from the true lumen and demonstrated homogeneous enhancement, while the left renal artery filled retrogradely from the false lumen, which remained partially supplied via the celiac inflow. These findings confirmed effective aortic sealing and restoration of visceral perfusion, despite the persistence of a limited distal false-lumen channel supplying the left renal artery.

Neurologically, sensory deficits resolved completely by postoperative day 2, with full recovery of proprioception and ambulation without assistance. Hemodynamic parameters stabilized without the need for vasopressors, and no procedure-related complications occurred (Table 1).

The patient continued to improve and was discharged on postoperative day seven, with preserved peripheral perfusion, normal neurological examination, and adequate renal function recovery.

## 3. Discussion

Type B aortic dissection (TBAD) requires therapeutic decisions based on the phase of disease and the presence of complications. The subacute period (15–90 days after onset) remains the most favorable window for TEVAR, as the aortic wall and intimal flap preserve remodeling potential, while procedural risks are lower compared with the hyperacute or chronic phases [3,9,10]. Our case—presenting approximately 8 weeks after symptom onset—fits precisely into this therapeutic window, and the favorable clinical evolution is consistent with current evidence.

The most distinctive feature in this case was the near-complete true-lumen collapse extending from the thoracic to the abdominal aorta, resulting in renal and visceral malperfusion as well as early spinal cord ischemia. This anatomical morphology represents one of the most challenging TBAD variants. In such scenarios, favorable aortic remodeling is strongly dependent on sealed proximal entry and true-lumen re-expansion, rather than on medical management alone. A severely narrowed true lumen has been repeatedly associated with persistent false-lumen patency, technical difficulty during device advancement, and inferior long-term outcomes [7,8,11,12,13,14,15,16,17,18,19]

As summarized in Table 2, several representative reports demonstrate that endovascular therapy remains effective even in anatomically hostile TBAD. Zhang et al. described restoration of flow and favorable remodeling in chronic TBAD with proximal true-lumen collapse after TEVAR, while Choo et al. confirmed in a clinical series that sealing the entry tear in slit-like true lumens promptly resolves malperfusion. Panesar et al. and Massmann et al. highlighted the feasibility of secondary endovascular approaches—fenestration or distal stent extension—in addressing distal lumen collapse or false-lumen deployment, restoring organ perfusion when initial outcomes were suboptimal. In cases with neurological compromise, Kim et al. reported improvement of spinal cord ischemia after fenestration, suggesting reversibility of early deficits once the true lumen is re-established. From a remodeling perspective, Le Huu et al. demonstrated that proximal sealing supported by selective distal scaffolding enhances true-lumen diameter and controls malperfusion, and Singh et al. emphasized that TEVAR-based strategies—whether isolated or scaffold-augmented—represent the most reliable framework for re-expanding the true lumen across TBAD morphologies.

Our patient displayed a combination of features reflected in these observations: extensive true-lumen collapse, multiorgan malperfusion, and a maturing intimal flap. A two-stage strategy was therefore required. First, the carotid–subclavian bypass secured posterior cerebral and left upper-limb perfusion while creating a safe proximal landing zone. Second, reverse-wire TEVAR restored physiologic flow through the true lumen, resulting in rapid normalization of hepatic and renal markers and full resolution of neurological symptoms. This sequential approach aligns with contemporary hybrid management principles for anatomically complex TBAD.

Beyond the anatomical considerations, the technical success of TEVAR also depends on the mechanical properties of the endografts used and their interaction with the dissected aortic wall. Current literature indicates that stent grafts with high conformability are preferable in the proximal arch, where inadequate apposition increases the risk of type Ia endoleak, whereas higher radial force devices are particularly advantageous in subacute or chronic dissections with stiffened intimal flaps and slit-like lumens [7,11,27,28]. In this context, a single-device strategy may fail to achieve both adequate arch sealing and distal re-expansion, especially in extensive anatomical involvement. Our approach, therefore, reflected a balance between proximal sealing safety and distal mechanical performance, tailored to the morphology of the dissection.

A fully endovascular alternative using a branched or fenestrated thoracic endoprosthesis was deliberately avoided. The combination of severe circumferential true-lumen collapse and marked false-lumen dominance would have made accurate endograft orientation and branch catheterization extremely challenging, increasing the risk of inadvertent false-lumen entry, distal maldeployment or type I endoleak. Additionally, given the patient’s evolving multiorgan malperfusion and early spinal ischemia, rapid and robust revascularization of the left subclavian territory was required, both to maintain vertebrobasilar perfusion and to ensure a stable proximal landing zone for TEVAR. A surgical carotid–subclavian bypass offered immediate and predictable flow restoration, whereas branched devices would have introduced procedural delays and higher technical uncertainty in an anatomically hostile aorta. Therefore, the staged hybrid strategy was selected to balance procedural safety, luminal re-expansion reliability and urgent organ-preservation needs.

Overall, this case reinforces that timely subacute intervention is pivotal, especially when malignant morphological markers are present. TEVAR—combined with selective adjunctive techniques—remains the most efficient means to depressurize the false lumen, restore visceral perfusion, and achieve rapid symptomatic recovery in patients with severe true-lumen collapse (Table 1). Fresilli et al. [29] recently proposed the *E-STABILISE* concept, combining transcatheter electrosurgical septotomy with the classic STABILISE maneuver to actively reconstruct the true lumen and deflate the false lumen in post-dissection aneurysms. Their approach demonstrates that targeted intimal disruption can be favorable in highly rigid chronic flaps, offering an alternative when conventional TEVAR alone does not adequately restore luminal geometry or distal perfusion.

Regarding limitations, this report describes a single case and does not include long-term imaging data. Although short-term results were excellent, persistent false-lumen perfusion remains a concern in this anatomical phenotype. Larger cohorts and standardized protocols are needed to determine which adjunctive strategies (fenestration, PETTICOAT, distal stent-grafting) yield the most favorable sustained remodeling.

The uniqueness of this case resides in the simultaneous presence of three high-risk features rarely encountered in subacute TBAD: (1) near-complete circumferential true-lumen collapse spanning the entire descending thoracic and abdominal aorta, (2) asymmetric visceral and renal malperfusion with SMA and celiac arising from a critically narrowed true lumen while the right renal artery was perfused by the false lumen, and (3) early spinal cord ischemia manifesting as bilateral proprioceptive deficit, despite preserved macro-peripheral pulses. This constellation is seldom described in the literature, which commonly reports isolated malperfusion phenotypes or chronic-phase lumen collapse, but not their simultaneous occurrence in the subacute window. Moreover, the technical approach—a staged hybrid pathway with carotid–subclavian revascularization followed by reverse-wire TEVAR employing differential stent-graft mechanics (arch conformability vs. distal radial force)—was specifically tailored to restore central flow in an anatomically hostile aorta and resulted in rapid neurological and organ function recovery. Current international guidelines, including the European Society for Vascular Surgery recommendations, acknowledge that complicated TBAD requires individualized endovascular management but provide no standardized protocol for patients with multi-territorial malperfusion combined with extensive true-lumen collapse, particularly in the subacute phase, where prosthesis behavior is highly dependent on flap stiffness and radial compression [30]. In this context, our case offers clinically actionable insight: it demonstrates that conventional “single-device TEVAR” may be insufficient, and that targeted hybrid sequencing coupled with device-specific biomechanical reasoning can decisively alter patient trajectory. We therefore believe that the relevance of this case extends beyond its narrative value—providing a pragmatic framework for clinicians faced with anatomically hostile TBAD variants, where real-time decision-making and procedural adaptability, rather than rigid algorithms, determine patient survival.

## 4. Conclusions

This case highlights the clinical complexity and therapeutic challenges associated with subacute type B aortic dissection complicated by severe true-lumen compression and multiorgan malperfusion. Early recognition of adverse prognostic features—including visceral, renal, and spinal cord hypoperfusion—was essential for timely intervention. A hybrid strategy combining left carotid–subclavian bypass with subsequent TEVAR proved safe and effective, restoring adequate true-lumen perfusion and leading to rapid clinical recovery. This experience reinforces the importance of tailored management strategies and timely endovascular repair during the subacute window, particularly in anatomically complex TBAD. Long-term imaging surveillance remains critical given the persistent risk of false-lumen progression.

## Figures and Tables

**Table 1:** Clinical, laboratory and imaging parameters before and after TEVAR.

	Pre-TEVAR	Early Post-TEVAR (48–72 h)	At Discharge
Arterial BP	Right arm: 150/80 mmHg Left arm: 125/50 mmHg	Stable, symmetric, no interarm gradient	130/70 mmHg
Heart rate	110 bpm	80–88 bpm	72 bpm
Serum creatinine	6.0 mg/dL	3.4 mg/dL	1.8 mg/dL
Urea	145 mg/dL	78 mg/dL	47 mg/dL
eGFR	<10 mL/min/1.73 m^2^	~25 mL/min/1.73 m^2^	~45 mL/min/1.73 m^2^
AST	86 U/L	52 U/L	39 U/L
ALT	92 U/L	58 U/L	48 U/L
Lactate	2.8 mmol/L	<2 mmol/L	Normal
CRP	18 mg/L	12 mg/L	5 mg/L
Sensory deficits	Bilateral lower limb numbness, impaired proprioception	Symptoms resolved	Normal
Coordination	Intermittent gait imbalance	Fully restored	Normal
True lumen	Severely compressed along descending thoracic and abdominal aorta	Re-expanded across thoracic segment	Stable re-expansion
Visceral perfusion	SMA and celiac from severely collapsed TL; R renal from FL	TL restored at visceral segment; R renal improved inflow	Full visceral patency; no endoleak
Procedure-related	—	No complications	No complications

**Table 2:** Publication reporting TBAD with severe true lumen (TL) compression.

Author.	Year	Publication Type	Management Strategy	Outcome/Key Findings
Zhang L. [20]	2021	Case report	TEVAR	Restoration of TL flow; favorable remodeling and clinical improvement
Choo S.J. [21]	2011	Clinical series	TEVAR for complicated TBAD with slit-like true lumen	Successful sealing of entry tear; improved TL perfusion; malperfusion resolution
Panesar H. [22]	2025	Case report	Endovascular reintervention (fenestration/distal stent extension) for distal TL collapse post-TEVAR	Resolution of malperfusion; re-expansion of distal TL
Massmann A. [23]	2024	Case report	Septal fenestration and TEVAR revision	Restored TL perfusion; normalization of visceral perfusion
Kim H. [24]	2020	Case report	Endovascular fenestration	Improvement of monoplegia; restored spinal cord perfusion
Le Huu A. [25]	2021	Clinical study	TEVAR ± distal bare-metal stents	Positive remodeling; improved TL diameter; controlled malperfusion
Singh S. [26]	2021	Expert analysis	TEVAR ± scaffolding stents	TEVAR advocated in all TBAD; scaffold stenting effective

## Data Availability

The raw data supporting the conclusions of this article will be made available by the authors on request.
